# Editorial: Recurrent fever in pediatrics

**DOI:** 10.3389/fped.2024.1500769

**Published:** 2024-10-17

**Authors:** Carla Gaggiano, Mohamed Tharwat Hegazy, Luca Cantarini, Gaafar Ragab

**Affiliations:** ^1^ Rheumatology Unit, Department of Medical Sciences, Surgery, and Neurosciences, University of Siena, Policlinico “Le Scotte”, Siena, Italy; ^2^ Rheumatology and Clinical Immunology Unit, Internal Medicine Department, Faculty of Medicine, Cairo University, Cairo, Egypt

**Keywords:** fever, recurrent, pediatrics, autoinflammatory diseases (AID), FMF

**Editorial on the Research Topic**
Recurrent fever in pediatrics

Recurrent fever poses a significant challenge in pediatric medicine, positioned at the confluence of molecular sciences, clinical specialties, and epidemiological research. Clinically, its management often necessitates a multidisciplinary approach to exclude potential infectious, immune-mediated, and hematologic-oncological etiologies. Recent research efforts in both pediatric and adult rheumatology have increasingly focused on recurrent fever syndromes including multifactorial and monogenic autoinflammatory diseases (1, 2). These syndromes, involving primarily innate immune system dysregulation, are characterized by recurrent, unprovoked febrile episodes usually starting during the pediatric age. They are associated with considerable morbidity, adversely affecting the physical and psychological well-being of children and their families. The research topic “*Recurrent fevers in pediatrics*” brings together a collection of articles that provide valuable insights into the molecular pathogenesis, diagnostic challenges, and therapeutic advancements related to recurrent fever syndromes.

The narrative review by Chaaban et al. delves into the complex cytokine networks implicated in familial Mediterranean fever (FMF), with a particular emphasis on the role of cytokines in the pathogenesis, disease susceptibility, clinical severity, and the development of targeted treatments in pediatric patients. The review provides an in-depth analysis of interleukin (IL)-1β, IL-1RA, IL-18, IL-6, tumor necrosis factor (TNF)-α, IL-4, macrophage migration inhibitory factor (MIF), vascular endothelial growth factor (VEGF), chemokines, and the S100A protein family. They stress the need for future studies comparing a broader cytokine spectrum across patients’ demographic and environmental factors. This review highlights the transformative impact of anti-cytokine therapies, particularly IL-1 inhibitors, in managing colchicine-resistant FMF, especially in pediatric patients with more severe and refractory disease courses. However, the review also acknowledges the limitations of these therapies, including challenges in discontinuing colchicine and the limited efficacy of TNF-α and IL-6 blockers.

The case report by Li et al. on Tumor Necrosis Factor Receptor-Associated Periodic Syndrome (TRAPS) provides crucial insights into the clinical and genetic challenges of diagnosing and managing rare recurrent fever syndromes. TRAPS is less prevalent than FMF and presents significant diagnostic difficulties due to its clinical overlap with other diseases. The report narrates the diagnostic journey of a pediatric patient with TRAPS, who experienced recurrent febrile episodes over several years before a definitive diagnosis was reached through the identification of the pathogenic C55Y mutation in the TNFRSF1A gene—a mutation that had not been previously reported in Asia. The authors highlight the importance of incorporating comprehensive genetic analysis early in the diagnostic workup of children with recurrent fever, especially when the clinical presentation is atypical or resistant to standard treatments.

In the context of multifactorial autoinflammatory diseases, this collection contributes valuable evidence to the current management strategies for periodic fever, aphthous stomatitis, pharyngitis, and adenitis (PFAPA) syndrome. Two studies stand out for addressing a critical gap in PFAPA management, where treatment options are primarily limited to glucocorticoids and, in select cases, tonsillectomy. Notably, a prospective clinical trial by Kapustova et al. investigates the use of ketotifen as a long-term prophylaxis for PFAPA syndrome, offering critical insights into the repurposing of existing drugs for managing autoinflammatory conditions. Ketotifen appears to significantly extend attack-free intervals in PFAPA patients, suggesting that this first-generation antihistamine, recognized for its immunomodulatory effects, could be a cost-effective and accessible treatment option for children with PFAPA syndrome worldwide. Moreover, the observational study by Spagnolo et al. investigates the use of Streptococcus salivarius K12 (SSK12) as a probiotic intervention to reduce the frequency and severity of febrile episodes in children with PFAPA syndrome. The findings that SSK12 significantly decreases the frequency of febrile episodes and alleviates most associated symptoms position this probiotic as a promising adjunctive therapy for PFAPA syndrome. Additionally, the study lends indirect support to the immunomodulatory effects of SSK12, likely attributed to its ability to colonize the oropharynx and competitively inhibit pathogenic microorganisms that may trigger inflammatory responses. This research aligns with the hypothesis that non-pharmacological interventions could be effective in managing PFAPA syndrome and potentially other autoinflammatory syndromes, particularly those in which gut microbiota and mucosal immunity are implicated.

Finally, a study conducted by Mansfield et al. on behalf of the Childhood Arthritis and Rheumatology Research Alliance (CARRA) PFAPA/Autoinflammatory Disease Working Group offers a unique epidemiological perspective on the interplay between the pandemic environment, external interventions, and the recognition, and possibly the clinical expression, of autoinflammatory diseases. The significant increase in the number of pediatric patients evaluated for recurrent fevers and autoinflammatory diseases in North America during the COVID-19 pandemic, compared to the previous year, suggests that public health measures—such as social distancing, which consequently reduced the spread of common childhood infections, along with heightened surveillance of fevers—may have contributed to greater awareness and earlier diagnosis of recurrent fever syndromes. However, the possibility of a direct trigger of post-infectious immune dysregulation by SARS-CoV-2 cannot be excluded, as evidenced by the emergence of Multisystem Inflammatory Syndrome in Children (MIS-C).

In conclusion, recent research on recurrent fever in pediatrics underscores the evolving understanding of these complex syndromes. It highlights that successful diagnosis and management increasingly depend on integrating various approaches to address the multifaceted nature of these conditions effectively. This reflects a broader trend in medicine towards precision diagnosis and therapies tailored to individual patient's genetic and molecular profiles. Also, the exploration of probiotics and the repurposing of existing drugs indicates a growing emphasis on non-pharmacological interventions and accessibility in pediatric care. Together, these advancements signal a more personalized and comprehensive strategy in managing recurrent fever syndromes, with the goal of enhancing outcomes and quality of life for affected children.
